# Graphene-Integrated Dual-Channel q-BIC Metasurface for Actively Tunable and Self-Calibrated Mid-Infrared SO_2_ Sensing

**DOI:** 10.3390/s26216889

**Published:** 2026-10-30

**Authors:** Zhengshan Xu, Dashuai Xu, Yang He, Tonggang Zhao

**Affiliations:** 1 Beijing Key Laboratory of Space-Ground Interconnection and Convergence, Beijing University of Posts and Telecommunications, Beijing 100876, China; hyxzs@bupt.edu.cn (Z.X.); dashuaixu@bupt.edu.cn (D.X.); yhe@bupt.edu.cn (Y.H.); 2 School of Electronic Engineering, Beijing University of Posts and Telecommunications, Beijing 100876, China

**Keywords:** mid-infrared gas sensing, SO_2_ detection, q-BIC metasurface, graphene tuning, ratiometric self-calibration, refractive-index drift

## Abstract

Mid-infrared gas sensors exploit molecular vibrational absorption but remain vulnerable to source fluctuation, background refractive-index drift, and fabrication-induced spectral mismatch. We numerically propose a graphene-integrated dual-channel quasi-bound-state-in-the-continuum (q-BIC) metasurface for SO_2_ detection near 8.6 μm. Symmetry breaking in square–hollow silicon nanopillars produces two leaky resonances with distinct modal origins, denoted P1 and P2. P1 is aligned with the SO_2_ absorption band and serves as the sensing channel, whereas P2 provides an off-band reference. Graphene gating enables post-fabrication spectral alignment. At the graphene Fermi level $E_{F}=0.516\;\mathrm{eV}$, the refractive-index sensitivities of P1 and P2 are 2280 nm RIU^−1^ and 564.6 nm RIU^−1^, respectively. Under 1% source fluctuation, the ideal common-mode model predicts an approximately 17 dB signal-to-noise ratio (SNR) improvement over single-channel detection. The field-overlap model gives an uncalibrated limit of detection (LOD) of 46.1 ppm and a conditional value of 6.5 ppm under an illustrative rescaled noise budget with negligible additional calibration errors. A value of 4.61 ppm requires the total corrected equivalent absorption noise to reach $1.0\times 10^{-7}$. These assumption-dependent estimates are not experimentally measured detection limits. The proposed architecture provides a compact platform for active spectral alignment and dual-channel self-calibration in mid-infrared gas sensing.

## 1. Introduction

Sulfur dioxide (SO_2_) is a major air pollutant released from fossil-fuel combustion, metal smelting, and chemical production [1]. Its emission is associated with acid rain, secondary particulate formation, and respiratory health risks, which makes continuous monitoring important in industrial and environmental settings [2]. Conventional SO_2_ detection methods, including colorimetric methods, metal-oxide sensors, electrochemical sensors, and wet-chemical analysis, have been used in many scenarios, but they often require bulky instruments, consumable sensing elements, or regular calibration [3,4,5,6,7]. Optical detection in the mid-infrared region offers a different route because many gas molecules have characteristic vibrational absorption bands in this spectral range [8]. The SO_2_ absorption band near 8.6 μm provides a target for resonance-enhanced absorption sensing [9]. Infrared techniques such as non-dispersive infrared spectroscopy and photoacoustic spectroscopy have also been used for SO_2_ detection, but compact on-chip implementation remains an important motivation for resonant photonic sensors [10,11,12,13]. This requirement has motivated the use of resonant photonic structures that can enhance light–matter interaction within a compact footprint [14].

Fiber-optic photoacoustic sensors offer a complementary approach to gas detection by combining absorption-induced acoustic or mechanical responses with interferometric readout. Recent implementations employ tuning-fork enhancement, coupled acoustic and cantilever resonances, or multipass excitation to improve sensitivity and enable compact or multi-gas sensing [15,16,17]. For direct optical readout, metasurfaces instead concentrate electromagnetic fields near the sensing interface and encode changes in the surrounding medium in their spectral response.

Among different resonant mechanisms, quasi-bound states in the continuum (q-BICs) are particularly attractive for sensing [18,19,20]. A symmetry-protected BIC is ideally decoupled from free-space radiation and cannot be directly observed under normal incidence. When a controlled structural asymmetry is introduced, the bound mode becomes weakly radiative and appears as a narrow q-BIC resonance with a finite linewidth [21,22]. The resulting high-quality-factor (*Q*) resonance can increase the spectral slope and improve refractive-index sensitivity [23,24,25,26]. However, the same narrow linewidth also makes q-BIC sensors vulnerable to practical errors. A small deviation in nanostructure size, etching depth, or hole position may shift the resonance away from the target molecular absorption band [27,28]. In addition, most reported metasurface gas sensors rely on a single resonance peak as the sensing signal. Such a single-channel readout cannot readily distinguish gas-induced absorption from source-power fluctuation, detector drift, or background refractive-index changes caused by temperature and humidity [29,30].

Active spectral tuning and differential calibration provide possible solutions to these limitations. Graphene is well suited for mid-infrared tunable photonics because its surface conductivity can be changed by electrostatic gating [31,32,33]. When graphene is integrated with a resonant metasurface, the resonance wavelength can be adjusted after fabrication by changing the Fermi level. This offers a potential means of correcting spectral offsets within the accessible tuning range, although it cannot restore a quality factor degraded by fabrication-induced scattering or loss. For gas detection, however, active tuning alone is not sufficient. The measured signal may still contain common-mode intensity noise and background refractive-index drift. A second resonance placed outside the target absorption band can serve as a reference channel. If the two resonances share the same optical path but respond differently to gas absorption and ambient refractive-index variation, their combined response can be used to separate the target-gas signal from environmental perturbations. Ratiometric calibration can suppress the correlated component of source-intensity fluctuations, consistent with common-mode rejection in optical sensing [29,30]; its effectiveness depends on the correlation and relative fluctuation amplitudes of the two channels.

In this work, we numerically investigate a graphene-integrated dual-channel q-BIC metasurface for mid-infrared SO_2_ sensing. Asymmetric square–hollow silicon nanopillars support two resonances, P1 and P2, which serve as the absorption-sensitive and off-band reference channels, respectively. Graphene gating through an HfO_2_ dielectric and a patterned gold grid provides spectral tuning, while field maps and multipole analysis guide the electrode placement. The readout combines the P1/P2 intensity ratio with a background-index estimate from the P2 wavelength shift. Because ppm-level gas absorption produces only small changes in the directly simulated transmission, gas-response slopes and detection limits are projected using a field-overlap perturbation model with explicit noise assumptions. This separates the electromagnetic design and modeled noise rejection from the calibration and experimental validation needed to establish gas-detection performance.

## 2. Materials and Methods

### 2.1. Metasurface Geometry and Numerical Model

**Figure 1:**
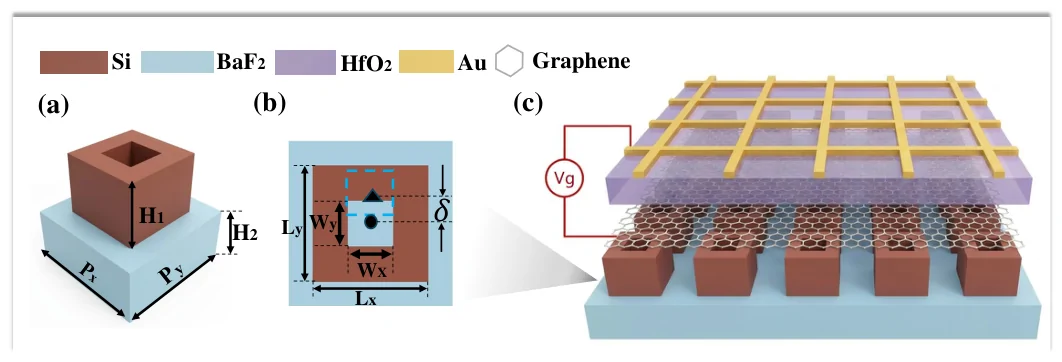
Schematic of the proposed metasurface sensor. (**a**) Three-dimensional view of the asymmetric square–hollow silicon nanopillar on a BaF_2_ substrate. *P* is the unit-cell period, $H_{1}$ is the silicon-pillar height, and $H_{2}$ is the BaF_2_-substrate thickness. (**b**) Top view of the unit cell, showing the outer side length of silicon *L*, hole side length *W*, and hole offset δ. (**c**) Full device configuration for SO_2_ sensing after integration of monolayer graphene, an HfO_2_ dielectric layer, and a patterned gold gate.

The proposed metasurface is composed of a periodic array of square hollow silicon nanopillars placed on a barium fluoride (BaF_2_) substrate, as shown in Figure 1a,b. Each unit cell has a period *P*. The silicon nanopillar has a height $H_{1}$ and an outer side length *L*. For the square unit cell, $P_{x}=P_{y}=P$, $L_{x}=L_{y}=L$, and $W_{x}=W_{y}=W$; directional subscripts in the figures refer to these same dimensions. The 1 μm-thick BaF_2_ layer $H_{2}$—which is transparent in the mid-infrared fingerprint region—is employed [34]. A square air hole with side length *W* is introduced inside the pillar. The surrounding-medium refractive index is denoted by *n*; n=1 specifies the ambient reference used in the passive and integrated spectra. Compared with a solid pillar, the hollow geometry modifies the modal field distribution and increases the dielectric–air interface of the resonator.

In-plane asymmetry is introduced by displacing the square hole from the center of the silicon pillar by a distance δ. This displacement breaks the mirror symmetry of the unit cell and opens a radiation channel for the otherwise symmetry-protected mode. Similar symmetry-breaking strategies have been widely used to transform ideal BIC modes into radiative q-BIC resonances with finite linewidths [35,36].

Numerical simulations were performed using the three-dimensional finite-difference time-domain method in Ansys Lumerical FDTD 2024 R1. Periodic boundary conditions were applied in the *x* and *y* directions to represent an infinite metasurface array, and perfectly matched layers were applied along the *z* direction. A normally incident plane wave propagated along the negative *z* direction, with its electric field polarized along the *x* axis. Transmission was monitored below the substrate to determine the resonance wavelengths, linewidths, and quality factors.

The complex optical constants of silicon were taken from the built-in “Si (Silicon)–Palik” material model [34]. The wavelength-dependent refractive index of BaF_2_ was obtained from the Li (1980) dataset in the refractiveindex.info database [37,38]. This dataset describes the refractive index at 293 K over 0.15 μm to 15 μm, covering the simulated 8 μm to 12 μm band. For the integrated sensing configuration used in Figure 7, a local mesh of 50 nm in the *x*, *y*, and *z* directions was used through the silicon and HfO_2_ regions. In the gold-grid region, the mesh was 50 nm in-plane and 5 nm along *z*.

Figure 1c shows the full sensing configuration after integration of monolayer graphene, an HfO_2_ dielectric layer, and a patterned gold gate electrode. These functional layers are introduced in Section 3, where the active spectral tuning and electrode design are discussed.

### 2.2. Symmetry Breaking and q-BIC Excitation

The square–hollow nanopillar supports symmetry-protected bound states when the air hole is centered in the silicon pillar. In this case, the unit cell preserves in-plane mirror symmetry, and the modal symmetry is incompatible with that of a normally incident plane wave. The corresponding resonant state is therefore decoupled from the external radiation channel and does not appear as a finite-width feature in the transmission spectrum. This state can be regarded as an ideal BIC in the lossless and infinitely periodic limit.

When the square hole is shifted by a finite offset δ, the in-plane symmetry is broken. The symmetry mismatch between the localized mode and the free-space radiation channel is partially removed, allowing the previously dark state to couple weakly to normally incident light. As a result, the ideal BIC is converted into a q-BIC with a finite radiative lifetime and a narrow spectral linewidth. The strength of this radiative coupling is controlled by the asymmetry parameter δ. A small δ gives weak leakage and a high *Q* factor, while a larger δ increases the coupling strength and broadens the resonance.

**Figure 2:**
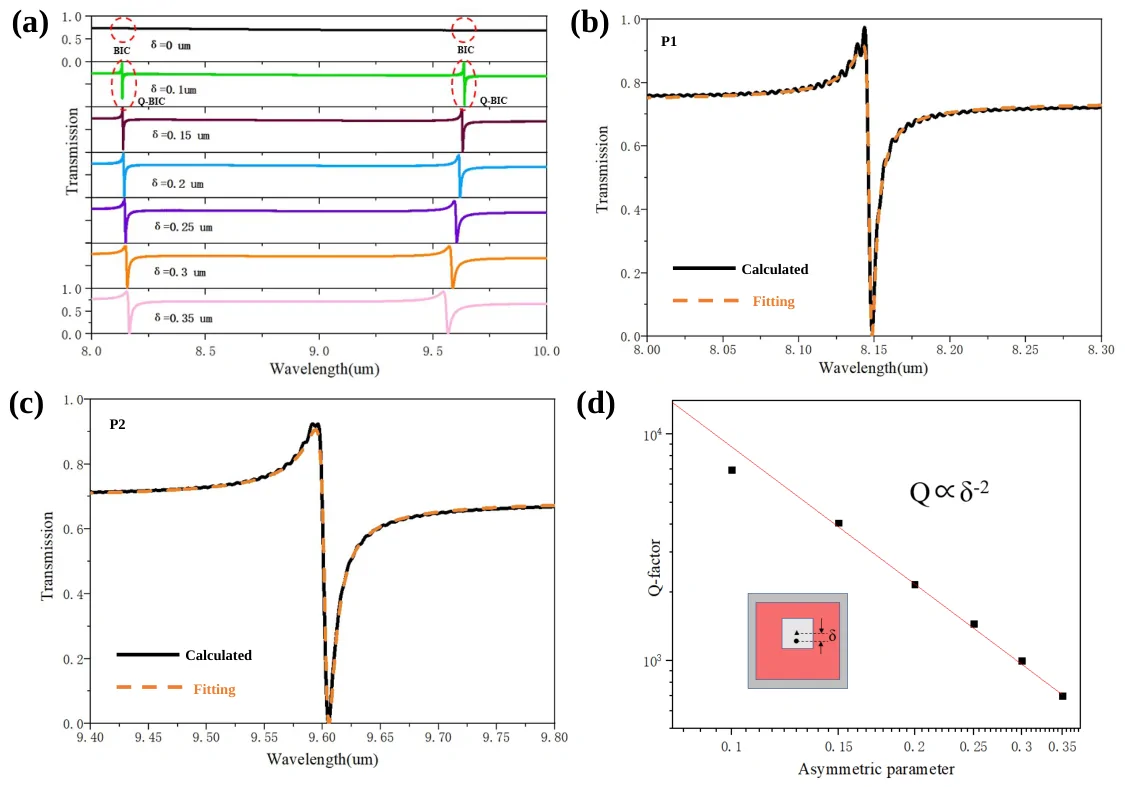
q-BIC excitation and Fano fitting of the asymmetric square–hollow silicon metasurface. ($P_{x}$ = $P_{y}$ = 5 μm, $H_{1}$ = 0.8 μm, $H_{2}$ = 1 μm, $L_{x}$ = $L_{y}$ = 4 μm and n = 1). (**a**) Transmission spectra as the hole offset δ is varied, showing the transition from the symmetry-protected BIC state to radiative q-BIC resonances. (**b**) Fano fitting of the P1 resonance. (**c**) Fano fitting of the P2 resonance. (**d**) Dependence of the P1 quality factor on the asymmetry parameter δ. The dashed line indicates the inverse-square scaling expected for symmetry-protected q-BIC modes.

Figure 2a shows the transmission spectrum ($P_{x}$ = $P_{y}$ = 5 μm, $H_{1}$ = 0.8 μm, $L_{x}$ = $L_{y}$ = 4 μm. $W_{x}$ = $W_{y}$ = 1.5 μm, and $H_{2}$ = 1 μm) of the Si-BaF_2_ metasurface under the vertical irradiation of incident light along the z-axis. For the symmetric structure (δ=0), no pronounced resonance is observed under normal incidence because the relevant modes remain symmetry protected. Once δ becomes nonzero, two sharp resonances appear in the mid-infrared region. The lower-wavelength resonance is P1, and the higher-wavelength resonance is P2. As δ increases, the two resonances remain narrow but their linewidths increase, indicating stronger radiative leakage from the q-BIC modes. At the same time, their resonance wavelengths shift in opposite directions: P1 shows a redshift, whereas P2 shows a blueshift. This different spectral evolution suggests that the two modes have different field distributions and modal origins, which are further examined by electromagnetic-field analysis and multipole decomposition in Section 3.

For the sensing design, P1 is selected as the signal resonance because it can be tuned close to the SO_2_ absorption band near 8.6 μm. P2 is located away from the SO_2_ absorption band and is therefore suitable as a reference resonance. This dual-resonance response is important for the later self-calibration scheme: the two peaks share the same optical path and system-level noise, but their spectral positions and modal sensitivities are different. The q-BIC mechanism therefore provides not only narrow linewidths for sensing, but also two spectrally separated channels for differential signal processing.

### 2.3. Fano Fitting and Q-Factor Evolution

The resonances generated by symmetry breaking show asymmetric spectral profiles, which are characteristic of Fano-type interference between a narrow discrete mode and a broad radiation continuum [39]. To quantify the resonance wavelength and linewidth, the transmission spectra of P1 and P2 were fitted using the Fano formula,

(1) $$T\left(\omega \right)=T_{0}+A_{0}\frac{{\left[q+2(\omega -\omega_{0})/\Gamma \right]}^{2}}{1+{\left[2(\omega -\omega_{0})/\Gamma \right]}^{2}},$$

where T(ω) is the transmission intensity, $T_{0}$ is the transmission offset, $A_{0}$ is the coupling amplitude, *q* is the Fano asymmetry parameter, $\omega_{0}$ is the resonance angular frequency, and Γ is the resonance linewidth. The quality factor is calculated as

(2) $$Q=\frac{\omega_{0}}{\Gamma }.$$

Figure 2b,c show the fitted spectra of P1 and P2 for the selected asymmetric structure. The fitting curves reproduce the simulated resonance profiles, confirming that both resonances originate from Fano-type coupling. For the bare square–hollow silicon metasurface, P1 is located at approximately 8.148 μm, while P2 appears at approximately 9.605 μm. The extracted *Q* factors are 1679 for P1 and 975 for P2, respectively. These values correspond to the passive dielectric metasurface before the integration of graphene, HfO_2_, and the gold gate electrode.

The evolution of the *Q* factor with the asymmetry parameter δ provides further evidence for the q-BIC origin of the resonances. In symmetry-protected q-BIC systems, the radiative loss is governed by the strength of the symmetry-breaking perturbation. For small perturbations, the radiative decay rate is proportional to $\delta^{2}$, and the radiation-limited quality factor follows:

(3) $$Q_{\mathrm{rad}}\propto \delta^{-2}.$$

This inverse-square scaling has been widely used as a signature of symmetry-protected q-BIC modes in all-dielectric metasurfaces. As shown in Figure 2d, the calculated *Q* factor of P1 decreases monotonically as δ increases and follows the expected $Q\propto \delta^{-2}$ trend. Physically, a larger offset opens a stronger radiation channel between the localized mode and free space, which increases the radiative leakage and broadens the resonance linewidth. At the same time, the resonance contrast becomes more pronounced because the mode couples more efficiently to the incident field.

This behavior illustrates the trade-off inherent to q-BIC sensing. A very small δ produces a high *Q* factor but weak external coupling and shallow spectral modulation. A larger δ improves the resonance contrast but reduces the *Q* factor. In the present design, δ is therefore chosen to maintain a narrow linewidth while ensuring sufficient modulation depth for transmission-based sensing. This balance is important because the subsequent gas detection relies not only on a high spectral slope but also on a stable and measurable resonance signal.

### 2.4. Geometric Tuning

In q-BIC metasurfaces, this requirement is more stringent than in broadband absorptive structures because the resonance linewidth is narrow. A small spectral deviation can reduce the overlap between the localized optical mode and the molecular fingerprint, thereby weakening the absorption-induced modulation.

**Figure 3:**
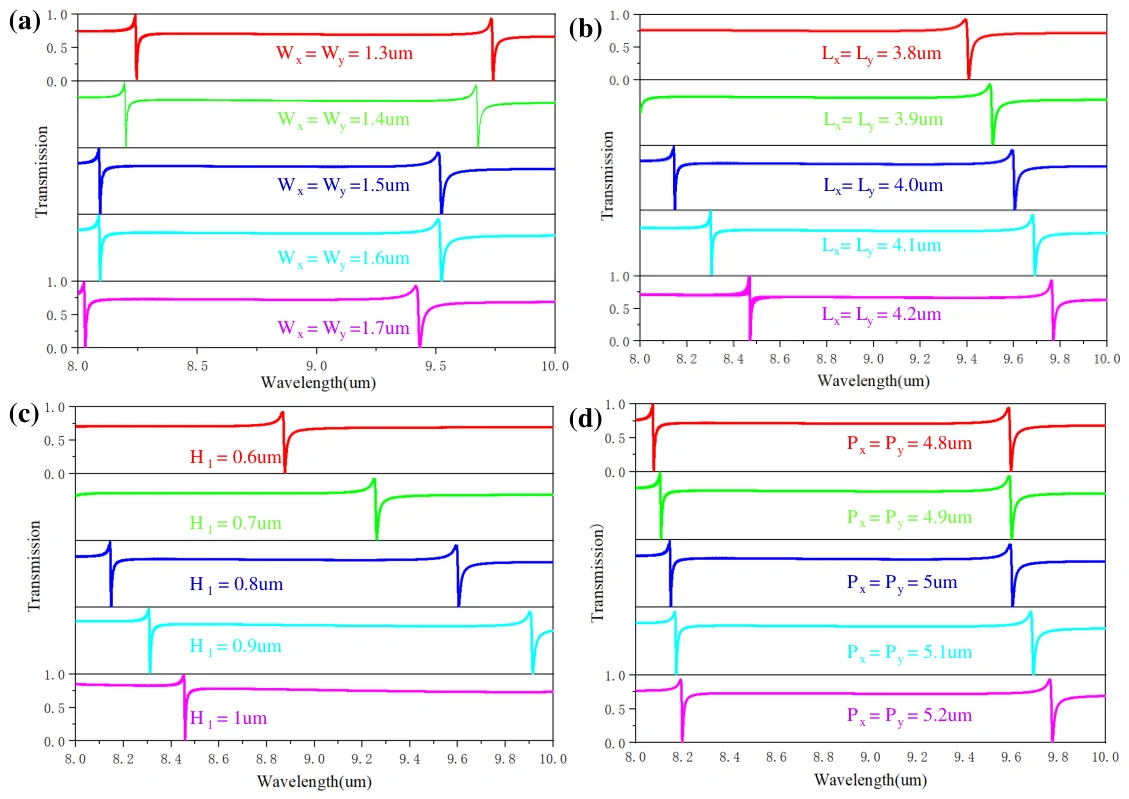
Dependence of the q-BIC resonances on geometric parameters of the square–hollow silicon metasurface. (δ = 0.25 μm, n = 1) (**a**) Transmission spectra for different hole side lengths *W* ($P_{x}$ = $P_{y}$ = 5 μm, $H_{1}$ = 0.8 μm, $H_{2}$ = 1 μm, $L_{x}$ = $L_{y}$ = 4 μm). (**b**) Transmission spectra for different outer side lengths *L* of the silicon pillar ($P_{x}$ = $P_{y}$ = 5 μm, $H_{1}$ = 0.8 μm, $H_{2}$ = 1 μm, $W_{x}$ = $W_{y}$ = 1.5 μm). (**c**) Transmission spectra for different pillar heights $H_{1}$ ( $P_{x}$ = $P_{y}$ = 5 μm, $H_{2}$ = 1 μm, $L_{x}$ = $L_{y}$ = 4 μm. $W_{x}$ = $W_{y}$ = 1.5 μm). (**d**) Transmission spectra for different lattice periods *P* ( $H_{1}$ = 0.8 μm, $H_{2}$ = 1 μm, $L_{x}$ = $L_{y}$ = 4 μm. $W_{x}$ = $W_{y}$ = 1.5 μm).

To evaluate the passive tuning capability of the proposed structure, the main geometric parameters were varied systematically, as shown in Figure 3. Increasing the side length of the central square hole *W* causes both P1 and P2 to shift toward shorter wavelengths. This blueshift can be attributed to the reduction of the effective optical path and modal volume inside the high-index silicon resonator. In contrast, increasing the outer side length *L* of the silicon pillar leads to a redshift of both resonances because the effective resonant cavity size becomes larger. A similar redshift is observed when the pillar height $H_{1}$ is increased, which is associated with the increased phase accumulation along the vertical direction. The lattice period *P* also affects the resonance wavelengths; a larger period shifts the resonances to longer wavelengths by modifying the inter-cell coupling and the effective radiation environment of the array.

The parameter sweeps show that P1 can be positioned near the SO_2_ absorption band while P2 remains in a relatively transparent spectral window. However, geometric tuning is available only at the design stage. Fabrication deviations in the hole size, pillar width, etching depth, sidewall profile, or lattice period may shift the narrow q-BIC resonances away from their intended wavelengths.

To correct such residual spectral mismatch after fabrication, the integrated device incorporates monolayer graphene as an electrically tunable layer, as described in Section 3.

## 3. Graphene Integration and Active Spectral Alignment

### 3.1. Integrated Graphene-Gated Sensor Architecture

Monolayer graphene is integrated with the passive metasurface to provide post-fabrication spectral control. Its gate-dependent surface conductivity in the mid-infrared region perturbs the effective optical environment of the q-BIC modes as the Fermi level $E_{F}$ is varied.

The integrated device configuration is shown in Figure 1c. From bottom to top, the structure consists of a BaF_2_ substrate, asymmetric square–hollow silicon nanopillars, a monolayer graphene sheet, an HfO_2_ dielectric layer, and a patterned gold gate electrode. The graphene layer is placed close to the resonant near-field region so that a change in its surface conductivity perturbs the effective optical environment of the q-BIC modes. The HfO_2_ layer acts as a gate dielectric and electrically separates graphene from the top gold electrode. The gate dielectric enables capacitive control of the graphene Fermi level, as used in graphene-based mid-infrared modulators [40].

The integrated architecture preserves the two resonance channels while enabling electrical adjustment of P1. P1 remains the absorption-sensitive resonance, whereas P2 serves as the off-band reference.

### 3.2. Field-Guided Design of the Patterned Gold Gate

Electrical tuning requires a conductive gate electrode, but metallic components can introduce ohmic absorption and degrade the linewidth of high-Q resonances [41]. This issue is particularly important for q-BIC metasurfaces because their sensing performance depends on low radiative and absorptive losses. The gate electrode must therefore be designed to provide a uniform bias to graphene while avoiding the regions where the resonant fields are strongly localized.

**Figure 4:**
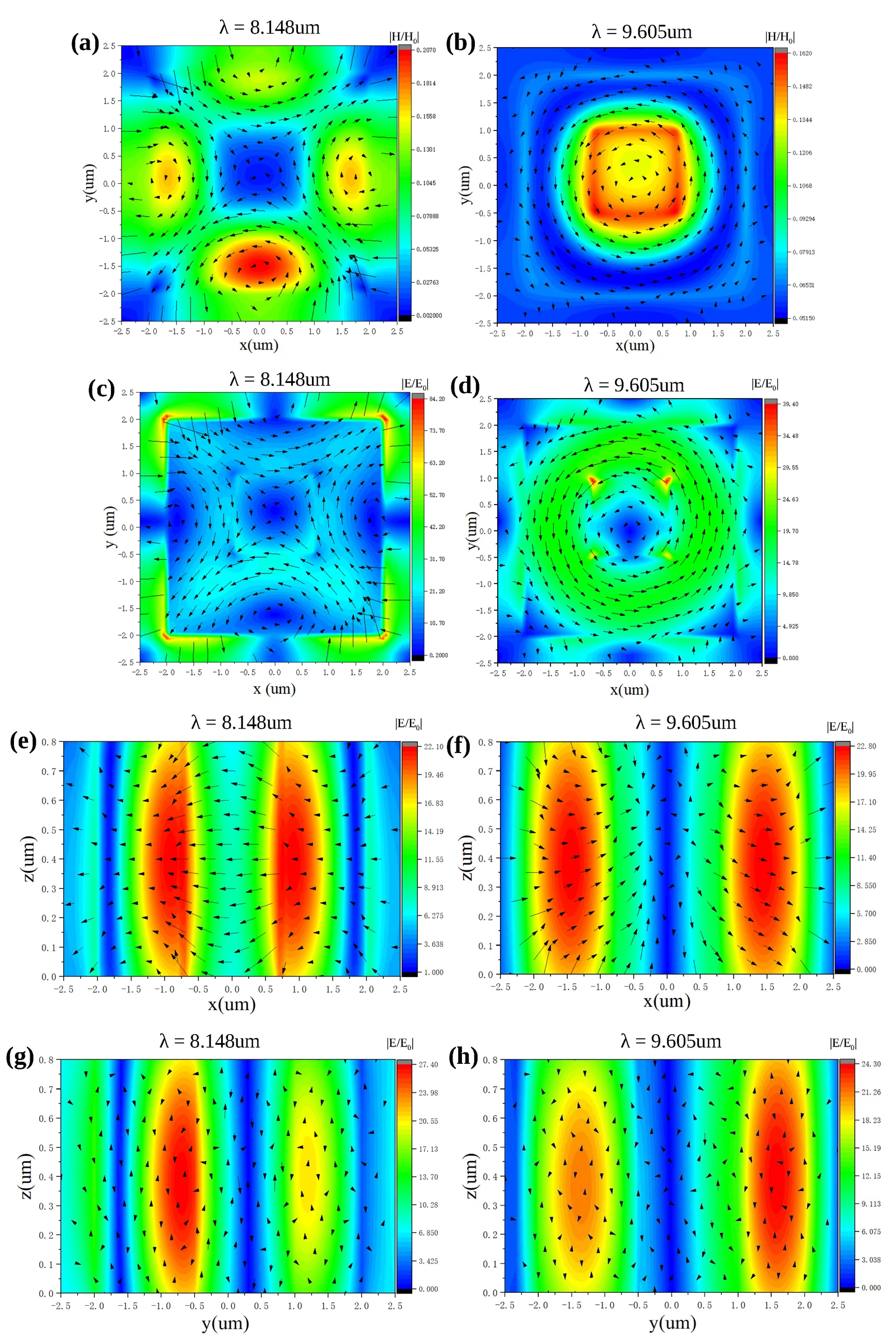
Electromagnetic-field distributions of the P1 and P2 resonances in the bare square–hollow silicon metasurface ($P_{x}$ = $P_{y}$ = 5 μm, $H_{1}$ = 0.8 μm, $H_{2}$ = 1 μm, $L_{x}$ = $L_{y}$ = 4 μm. $W_{x}$ = $W_{y}$ = 1.5 μm, δ = 0.25 μm and n = 1). (**a**,**b**) Magnetic-field distributions of P1 and P2 in the selected cross-sectional plane. (**c**,**d**) Electric-field distributions of P1 and P2 in the xy-plane. (**e**,**f**) Electric-field distributions of P1 and P2 in the xz-plane. (**g**,**h**) Electric-field distributions of P1 and P2 in the yz-plane. The arrows indicate the local electric-field direction.

To determine suitable electrode positions, the electromagnetic-field distributions of the bare silicon metasurface (comprising only BaF_2_ and silicon structures), were first examined at P1 and P2. As shown in Figure 4a,b, the magnetic-field distributions of the two modes are different. For P1, the field is distributed around the square hollow cavity and near the sidewalls of the silicon pillar. For P2, the magnetic field is more concentrated inside the hollow cavity. The electric-field maps in Figure 4c–h further show that both resonances have strong near-field confinement close to the silicon edges and the inner cavity boundary. These regions should be kept away from the metal gate to avoid additional absorption and resonance broadening.

**Figure 5:**
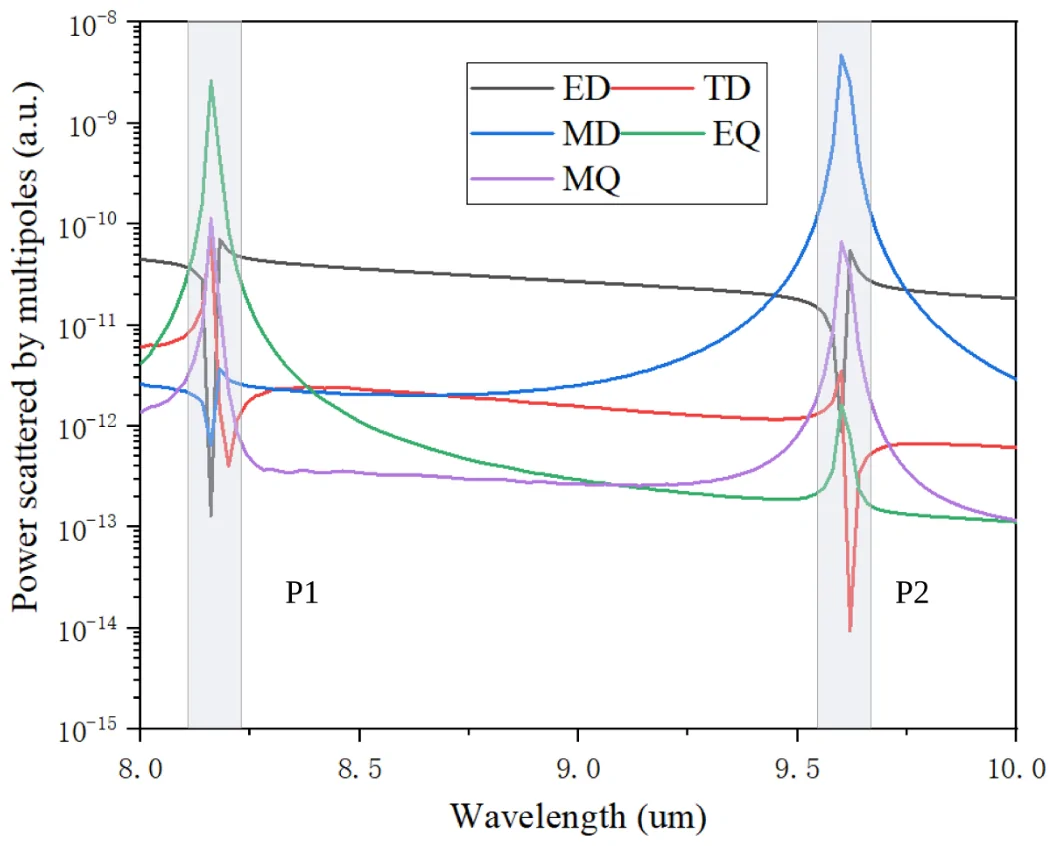
Cartesian multipole decomposition of the P1 and P2 resonances in the bare square–hollow silicon ($P_{x}$ = $P_{y}$ = 5 μm, $H_{1}$ = 0.8 μm, $L_{x}$ = $L_{y}$ = 4 μm. $W_{x}$ = $W_{y}$ = 1.5 μm, $H_{2}$ = 1 μm, δ = 0.25 μm and n = 1). The radiated powers of the electric dipole (ED), magnetic dipole (MD), toroidal dipole (TD), electric quadrupole (EQ), and magnetic quadrupole (MQ) components are calculated from the displacement-current distribution. P1 is mainly associated with an electric-quadrupole-like response, while P2 is dominated by a magnetic-dipole-like response.

Cartesian multipole decomposition was used to identify the modal origins of the two resonances. The radiated powers of the electric dipole (ED), magnetic dipole (MD), toroidal dipole (TD), electric quadrupole (EQ), and magnetic quadrupole (MQ) components were calculated from the displacement-current distribution [42,43,44]. The results in Figure 5 show that P1 is mainly associated with an electric-quadrupole-like response, whereas P2 is dominated by a magnetic-dipole-like response. This difference is consistent with the field distributions in Figure 4 and explains why the two resonances have different sensitivities to the surrounding medium.

Based on the field maps and multipole analysis, the gold electrode is designed as a hollow grid rather than a continuous film. The grid lines are placed in the low-field regions between adjacent unit cells. This arrangement reduces spatial overlap between the metal and the resonant near field while maintaining electrical continuity for graphene gating. The resulting structure provides the biasing path needed for active tuning and, at the same time, limits the loss-induced degradation of the q-BIC resonances. Additionally, the integrated architecture induces a redshift in both P1 and P2, bringing P1 closer to the SO_2_ absorption peak (8.6 μm) and thereby facilitating the design of the SO_2_ sensor.

### 3.3. Electrode-Width Optimization and Loss Analysis

Electrical gating requires the gold grid to have sufficient continuity and low sheet resistance. At the same time, the metal linewidth must be kept small to reduce the overlap between the electrode and the localized q-BIC fields. This trade-off is important because metallic components introduce ohmic absorption, which broadens the resonance linewidth and lowers the quality factor. With the metasurface geometric parameters set to $P_{x}$ = $P_{y}$ = 5.4 μm, $H_{1}$ = 1.1 μm, $L_{x}$ = $L_{y}$ = 4 μm. $W_{x}$ = $W_{y}$ = 1.5 μm, δ= 0.25 μm, $H_{2}$ = 1 μm and with the thicknesses of the gold (Au) layer ($H_{\mathrm{Au}}$) and the HfO_2_ layer ($H_{{\mathrm{HfO}}_{2}}$) both set to 50 nm, we optimized the electrode linewidth ($w_{\mathrm{Au}}$) by comparing three representative values: 100 nm, 200 nm, and 500 nm.

Table 1 summarizes the resonance wavelengths, *Q* factors, and baseline transmittance of the integrated graphene-gated metasurface for different $w_{\mathrm{Au}}$($E_{F}=0\mathrm{eV}$). Here $\lambda_{P1}$ and $\lambda_{P2}$ denote the P1 and P2 resonance wavelengths, $Q_{P1}$ and $Q_{P2}$ their quality factors, and $T_{\mathrm{base}}$ the baseline transmittance. When $w_{\mathrm{Au}}=100\;nm$, P1 is located at 8.649 μm and P2 at 10.744 μm. Both resonances retain *Q* factors of 456, and the baseline transmittance remains at 86.5%. Increasing the linewidth to 200 nm slightly reduces the transmittance and lowers the *Q* factors to 397 for P1 and 411 for P2. When $w_{\mathrm{Au}}$ is further increased to 500 nm, the degradation becomes more pronounced: the *Q* factor decreases to 263 for P1 and 213 for P2, while the baseline transmittance drops to 83.7%.

This trend is consistent with the field-guided design discussed in Section 3.2. A wider gold line extends closer to the high-field regions near the silicon sidewalls and the hollow cavity boundary. The increased metal–field overlap strengthens ohmic damping and reduces the stored resonant energy. As a result, the resonance linewidth broadens and the *Q* factor decreases. The resonance wavelength also changes slightly with electrode linewidth, indicating that the gold grid not only introduces absorption loss but also perturbs the effective optical environment of the metasurface.

Based on these results, $w_{\mathrm{Au}}=100\;nm$ is selected for the final device. This linewidth limits optical loss while maintaining an electrically connected gate for graphene biasing. The corresponding *Q* factor is lower than that of the bare dielectric q-BIC metasurface discussed in Section 2.3, where P1 reaches Q=1679. This reduction is expected because the integrated device includes graphene, HfO_2_ and a metallic gate. Nevertheless, the integrated structure still provides narrow resonances, high baseline transmission, and a sufficient spectral slope for transmission-based SO_2_ sensing. The comparison also shows that a continuous or excessively wide metal electrode is not suitable for the present q-BIC sensor.

### 3.4. Graphene-Enabled Spectral Locking to the SO_2_ Band

**Figure 6:**
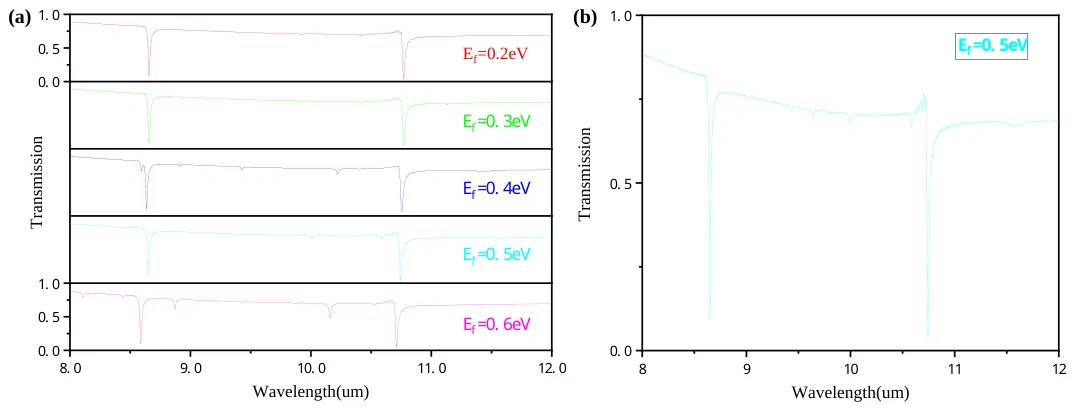
Active spectral tuning of the integrated graphene-gated q-BIC metasurface ($P_{x}$ = $P_{y}$ = 5.4 μm, $H_{1}$ = 1.1 μm, $L_{x}$ = $L_{y}$ = 4 μm. $W_{x}$ = $W_{y}$ = 1.5 μm, δ= 0.25 μm, $H_{2}$ = 1 μm, $H_{{\mathrm{HfO}}_{2}}$ = 50 nm and n = 1). (**a**) Transmission spectra under different graphene Fermi levels $E_{F}$, showing the electrically induced blueshift of P1 and P2. (**b**) Transmission spectrum at the representative aligned state of $E_{F}=0.5\mathrm{eV}$, where P1 is aligned with the SO_2_ absorption band near 8.6 μm and P2 remains in the reference spectral window near 10.7 μm.

Figure 6a shows the transmission spectra of the integrated metasurface as $E_{F}$ is varied. The $E_{f}$ labels in the figure denote the graphene Fermi level $E_{F}$. Both resonances shift toward shorter wavelengths with increasing Fermi level, indicating a reduction in the modal effective index. This tuning range can compensate for a fabrication-induced redshift of the sensing resonance.

At the representative state $E_{F}=0.5\;\mathrm{eV}$ shown in Figure 6b, P1 lies close to the SO_2_ absorption band near 8.6 μm, while P2 remains near 10.7 μm. Refining the Fermi level to $E_{F}=0.516\;\mathrm{eV}$ places P1 at 8.6016 μm and P2 at 10.7228 μm. At this locked sensing state, the P1 quality factor extracted from the simulated spectrum is Q=506.86.

The locked state is used for the refractive-index calibration, field-overlap analysis, and LOD calculation in Section 4. It is distinct from the electrode-linewidth comparison state in Table 1, for which Q=456 is reported. Although graphene, HfO_2_, and the gold grid introduce additional optical loss, the integrated device retains resolvable resonances and adequate transmission. The relevant design trade-off is therefore between post-fabrication spectral correction and linewidth broadening, rather than the maximization of *Q* alone.

## 4. Dual-Channel Sensing and Ratiometric Self-Calibration

### 4.1. Dual-Channel Correction of Background Refractive-Index Drift

The active spectral alignment described in Section 3 places P1 close to the SO_2_ absorption band and leaves P2 in a reference spectral window. In the present readout, SO_2_ concentration is quantified from the absorption-induced intensity response of P1 rather than from a concentration-induced wavelength shift. The wavelength responses of P1 and P2 are instead used to estimate and correct background refractive-index drift so that the sensing resonance remains at a defined spectral operating point.

Multi-parameter optical sensors have previously used two spectral features with different response coefficients to decouple refractive-index and temperature changes [45]. The present design adopts a related dual-observable principle but assigns different functions to the two q-BIC resonances: P2 tracks the background refractive-index drift, whereas P1 provides the absorption-sensitive readout.

Let $\lambda_{Pi}$ and $\lambda_{Pi}^{\left(0\right)}$ be the current and reference resonance wavelengths of channel *i* (i=1,2), respectively, with $n_{0}$ denoting the reference surrounding-medium index. The total shift is $\Delta \lambda_{i}=\lambda_{Pi}-\lambda_{Pi}^{\left(0\right)}$. For small background-index perturbations, the wavelength shifts can be written as

(4) $$\left[\begin{array}{c} \Delta \lambda_{1}\\ \Delta \lambda_{2} \end{array}\right]=\left[\begin{array}{c} S_{1,n}\\ S_{2,n} \end{array}\right]\Delta n+\left[\begin{array}{c} \delta \lambda_{1}\\ \delta \lambda_{2} \end{array}\right],$$

where $S_{i,n}$ is the refractive-index sensitivity of channel *i*, $\Delta n=n-n_{0}$ is the background refractive-index variation, and $\delta \lambda_{i}$ is the residual wavelength shift not described by the linear background-index term. Because P2 is outside the main SO_2_ absorption band, its shift $\Delta \lambda_{2}$ provides the reference estimate

(5) $$\hat{\Delta n}=\frac{\Delta \lambda_{2}}{S_{2,n}}.$$

The corresponding index-corrected P1 shift $\Delta \lambda_{1,\mathrm{corr}}$ is

(6) $$\Delta \lambda_{1,\mathrm{corr}}=\Delta \lambda_{1}-\left(\frac{S_{1,n}}{S_{2,n}}\right)\Delta \lambda_{2}.$$

Equation (6) removes the estimated index-induced displacement of P1. It is used for spectral tracking and operating-point correction only; it is not used as the SO_2_ concentration estimator. The detailed derivation of Equations (4)–(6), including the definition of the residual term $\delta \lambda_{i}$ and the approximation $\delta \lambda_{2}\approx 0$ used in Equation (5), is provided in Section S1 of the Supplementary Information.

### 4.2. Extraction of Refractive-Index Sensitivities

The refractive-index sensitivities of P1 and P2 were extracted from simulations of the integrated device at the final sensing state of $E_{F}=0.516\;\mathrm{eV}$. The surrounding refractive index *n* was varied from 1.00 to 1.05, and the resonance wavelengths $\lambda_{P1}$ and $\lambda_{P2}$ are listed in Table 2.

Both resonances show nearly linear redshifts as the surrounding refractive index increases. P1 shifts from 8.6016 μm to 8.7152 μm, and a linear fit gives a sensitivity of 2280.0 nm RIU^−1^. P2 shifts from 10.7228 μm to 10.7512 μm, and a linear fit gives a lower sensitivity of 564.6 nm RIU^−1^. The coefficients of determination are $R^{2}=0.9999$ for P1 and $R^{2}=0.9991$ for P2, supporting the linear index-correction model within this range.

The different refractive-index sensitivities are consistent with the modal analysis in Section 3.2. P1 has stronger field exposure near the hollow cavity and the silicon sidewalls, where the surrounding medium perturbs the effective modal index more strongly. P2 is less sensitive to the background index but remains sufficiently stable and measurable as a reference resonance. The sensitivity ratio $S_{1,n}/S_{2,n}$ is approximately 4.04, which provides the weighting factor used for refractive-index-drift correction in Equation (6).

The same simulations also show that the resonance depths vary with the surrounding index.

### 4.3. Ratiometric Suppression of Common-Mode Intensity Noise

The wavelength correction in Section 4.1 and Section 4.2 addresses background refractive-index drift. For the intensity readout, the measured P1/P2 ratio is defined as $R=I_{1}/I_{2}$, where $I_{1}$ and $I_{2}$ are the measured intensities at the P1 and P2 resonances, respectively. In the ideal ratiometric model, the two channels have identical fractional source fluctuations, which cancel to first order in their intensity ratio.

For the ideal common-mode model, Table 3 compares the calculated single-channel and ratiometric SNR values. Here $\sigma_{\epsilon }$ denotes the standard deviation of the fractional source-intensity fluctuation. At 1% source fluctuation, the ratiometric readout gives a calculated gain of 17.03 dB. This gain compares the total single-channel and ratio noise levels in the ideal model, including the residual detector noise; it is not a separate attenuation factor for the common-mode component. Fitting and baseline-calibration uncertainties contribute additionally.

The no-gas resonance depths also vary with the background refractive index. Therefore, a constant reference ratio cannot be used. The index-dependent no-gas baseline is defined as

(7) $$R_{0}\left(n\right)=\frac{T_{1,0}\left(n\right)}{T_{2,0}\left(n\right)},$$

where $T_{1,0}\left(n\right)$ and $T_{2,0}\left(n\right)$ are the no-gas transmission values at P1 and P2, respectively. Using the P2 wavelength estimate in Equation (5), the baseline is interpolated at $\hat{n}=n_{0}+\hat{\Delta n}$, where $\hat{n}$ is the estimated surrounding-medium index. The corrected equivalent absorption observable is

(8) $$A_{\mathrm{corr}}=-\ln\left[\frac{R}{R_{0}\left(\hat{n}\right)}\right].$$

For a weak SO_2_ perturbation, $A_{\mathrm{corr}}\approx S_{A}\Delta C$, where $S_{A}$ is the field-overlap absorption-response slope derived in Section 4.4 and ΔC is the concentration change relative to the no-gas baseline.

Sharing an optical path does not guarantee identical fractional fluctuations at P1 and P2. For small fluctuations, the first-order log-ratio variance is $\sigma_{\ln R}^{2}=s_{1}^{2}+s_{2}^{2}-2\rho s_{1}s_{2}+d_{1}^{2}+d_{2}^{2}$, where $s_{i}$ and $d_{i}$ are fractional source- and detector-noise standard deviations and ρ is the source-noise correlation coefficient. Detector noise is assumed independent between channels and uncorrelated with source noise. With $s_{1}=s_{2}=0.01$ and $d_{1}=d_{2}=0.001$, the calculated gain decreases from 17.03 dB at ρ=1 to 14.02 dB at ρ=0.99 and 9.25 dB at ρ=0.95. Even at ρ=1, unequal source fluctuations leave a residual variance of ${\left(s_{1}-s_{2}\right)}^{2}$. Interpreting noise rejection as a gas-detection SNR gain also assumes an unchanged concentration-response slope and negligible target response in P2. Resonance-fitting and baseline-calibration uncertainties contribute separately. The ratiometric noise model, Taylor expansion, and covariance analysis are given in Section S2 of the Supplementary Information.

### 4.4. Limit of Detection

Direct FDTD concentration sweeps at ppm-level SO_2_ concentrations yielded nearly overlapping transmission spectra at the available export precision and did not provide a resolved concentration-response slope. The LOD is instead estimated using a field-overlap absorption model under the weak-perturbation approximation. This model combines the simulated q-BIC field distribution with the SO_2_ absorption cross section obtained from the HITRAN database [46] and an assumed corrected absorption-noise level. The detailed field-overlap derivation and noise-assumption decomposition are given in Section S3 of the Supplementary Information.

**Figure 7:**
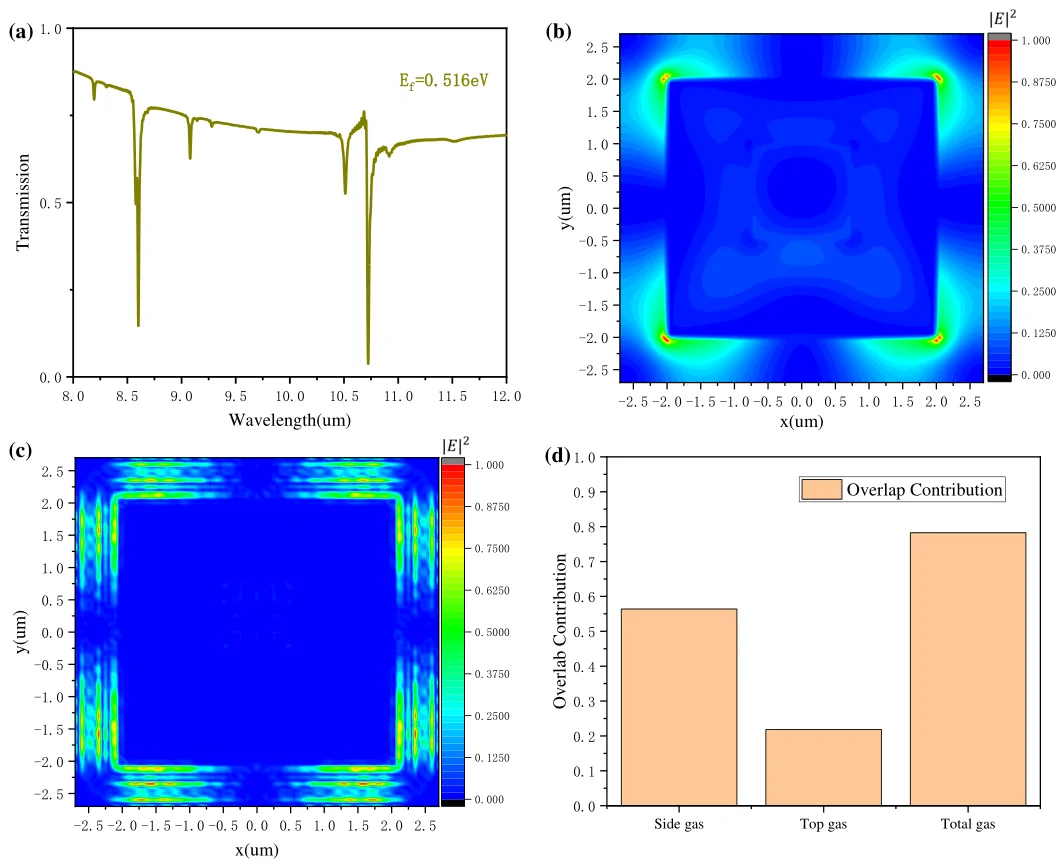
Field-overlap evidence for SO_2_ absorption at the aligned P1 resonance ($P_{x}$ = $P_{y}$ = 5.4 μm, $H_{1}$ = 1.1 μm, $L_{x}$ = $L_{y}$ = 4 μm. $W_{x}$ = $W_{y}$ = 1.5 μm, δ= 0.25 μm, $H_{2}$ = 1 μm, $H_{{\mathrm{HfO}}_{2}}$ = 50 nm and n = 1). (**a**) Transmission spectrum at $E_{F}=0.516\mathrm{eV}$, with P1 aligned near 8.6016 μm and P2 near 10.7228 μm. (**b**,**c**) Normalized ${\left|E\right|}^{2}$ distributions in the side-accessible and top gas regions. (**d**) Gas-overlap contributions: $\Gamma_{\mathrm{side}}=0.5640$, $\Gamma_{\mathrm{top}}=0.2185$, and $\Gamma_{\mathrm{gas}}=0.7826$.

At the final sensing state of $E_{F}=0.516\mathrm{eV}$, P1 is aligned at 8.6016 μm. Figure 7 shows the field-overlap result used in the LOD calculation. The total gas-overlap factor, defined from the normalized ${\left|E\right|}^{2}$ integral over the gas-accessible region, is $\Gamma_{\mathrm{gas}}=0.7826$, comprising $\Gamma_{\mathrm{side}}=0.5640$ from the side-accessible gas region and $\Gamma_{\mathrm{top}}=0.2185$ from the top region. The stronger sidewall and corner fields therefore provide the dominant SO_2_ interaction volume.

In the *Q*-based effective interaction length $L_{\mathrm{eff}}=\eta Q\lambda_{P1}/\left(2\pi n_{\mathrm{eff}}\right)$, η is the coupling/interaction-length factor and $n_{\mathrm{eff}}$ is the modal effective-index normalization; both are idealized, unmeasured parameters. Under the baseline normalization η=1 and $n_{\mathrm{eff}}=1$, the field-overlap calculation gives an equivalent absorption-response slope of $S_{A}=6.50\times 10^{-8}\;{\mathrm{ppm}}^{-1}$. The calculated LOD scales as $n_{\mathrm{eff}}/\eta$ if different interaction-length normalization factors are used. The LOD is evaluated from

(9) $$\mathrm{LOD}=\frac{3\sigma_{A}}{S_{A}},$$

where $\sigma_{A}$ is the standard deviation of the corrected equivalent absorption observable $A_{\mathrm{corr}}$.

For an uncalibrated equivalent absorption noise of $\sigma_{A,\mathrm{uncal}}=10^{-6}$, the field-overlap calculation gives ${\mathrm{LOD}}_{\mathrm{uncal}}=46.1\mathrm{ppm}$. A separate illustrative noise budget sets the fractional source noise to $s=9.9504\times 10^{-7}$ and the independent detector noise in each channel to $d=9.9504\times 10^{-8}$. With s/d=10, ρ=1 and negligible additional calibration errors, the single-channel total noise is $10^{-6}$ and the ratio noise is $\sqrt{2}d=1.4072\times 10^{-7}$, giving a conditional LOD of 6.5 ppm. The total-noise ratio remains 7.1063 (17.03 dB); this hypothetical rescaling does not establish an achieved absolute noise floor. Section S3 and Tables S2–S4 of the Supplementary Information specify the inputs, noise budgets and sensitivity analysis. If the total corrected equivalent absorption noise reaches $\sigma_{A,\mathrm{corr}}=10^{-7}$, the calculated LOD is 4.61 ppm.

The resulting field-overlap-based LOD calculations are summarized in Table 4. The table distinguishes the uncalibrated baseline, the illustrative rescaled ratio budget, and the specified total corrected-noise case.

At fixed $S_{A}$, a  20 increase in $\sigma_{A}$ raises the LOD by 20; at fixed $\sigma_{A}$, a  20 decrease or increase in $|S_{A}|$ changes the LOD by factors of 1.25 or 0.833, respectively. For small independent uncertainties in $\sigma_{A}$ and $|S_{A}|$, their relative variances add to give the relative LOD variance; correlated inputs require covariance terms. Uncertainties in the field overlap, gas-accessible volume, and interaction-length normalization enter through $S_{A}$. The detector-noise assumptions used for the illustrative 17.03 dB gain give $\sigma_{\ln R}=1.414\times 10^{-3}$ at ρ=1 and do not establish the total absorption-noise level of $10^{-7}$ assumed for the 4.61 ppm projection.

## 5. Discussion

The proposed architecture combines electrical alignment of the sensing resonance with a reference-assisted intensity readout in the 8 μm to 12 μm band. Table 5 summarizes related sensing approaches by spectral coverage, target analyte, detection mechanism, and evidence type. Refractive-index sensitivity characterizes the spectral response to changes in the surrounding medium, whereas a gas-detection limit depends on molecular absorption, optical interaction strength, and readout noise. These distinctions define the scope of quantitative comparisons between sensing platforms.

Meng et al. demonstrated a gas sensing microspectrometer operating over 7 μm to 14 μm, with a metasurface filter array integrated with a microbolometer camera [47]. Its demonstrated analytes include ammonia and methyl ethyl ketone (MEK). The overlapping spectral coverage illustrates the use of metasurfaces for mid-infrared gas analysis, with an optical arrangement and analytes different from those considered here. The fiber-optic photoacoustic studies summarized in Table 5 provide experimental examples of complementary gas-absorption readout mechanisms [15,16,17]. Related metasurface mechanisms include Fano-resonant refractive-index and methane sensing [48], graphene-based multiband absorption [49], and gas sensing through reversible chemical doping of graphene [50].

Integration of graphene, HfO_2_, and the gold grid introduces a trade-off between resonance linewidth and electrical tunability. Relative to the bare dielectric metasurface, the integrated structure has a lower *Q* factor while allowing electrical spectral alignment. At $E_{F}=0.516\mathrm{eV}$, the reported numerical refractive-index sensitivities are 2280 nm RIU^−1^ for P1 and 564.6 nm RIU^−1^ for P2, with a P1 *Q* factor of 506.9 at the same operating point. These quantities characterize the simulated optical response used in the sensing analysis.

The two channels address background-index drift and source-intensity fluctuations through distinct observables. The P2 shift $\Delta \lambda_{2}$ estimates the background refractive-index change, while the calibrated $R_{0}\left(n\right)$ baseline corrects the associated index dependence of the P1/P2 intensity ratio. Under the ideal common-mode model, this ratio gives a 17.03 dB SNR gain at 1% source fluctuation. The gain compares total noise levels within this model, including detector noise; fitting and calibration uncertainties contribute additionally.

Practical operation also requires consideration of temperature, humidity, and coexisting gases. Temperature changes can affect the readout through changes in material optical properties and gas absorption, while humidity may introduce water-vapor absorption and adsorption-related drift. These perturbations need not produce the same dual-channel response as a background-index change; P2-based correction therefore compensates only for contributions represented by the calibration model. Potential interferents, including H_2_O and SO_3_, should be assessed using the spectral responses of both channels at relevant temperatures, pressures, and concentrations. Temperature and humidity calibration, together with mixed-gas measurements, is needed to establish environmental robustness and practical selectivity.

The field-overlap absorption model gives an uncalibrated LOD of 46.1 ppm. A value of 6.5 ppm follows from the illustrative rescaled noise budget in Section S3, with total corrected noise of approximately $1.41\times 10^{-7}$ and negligible additional calibration errors, while 4.61 ppm requires a total corrected equivalent absorption noise of $10^{-7}$. All three estimates assume η=1 and $n_{\mathrm{eff}}=1$ and scale as $n_{\mathrm{eff}}/\eta$. They are conditional model estimates, not concentration limits directly resolved by FDTD sweeps or measured experimentally. The field-overlap derivation and noise assumptions are provided in Section S3 of the Supplementary Information.

The field-overlap treatment assumes that gas absorption is a weak perturbation of the resonant field. Its quantitative accuracy depends on the simulated field distribution and resonance parameters, the gas-accessible interaction volume, and the interaction-length normalization. Numerical uncertainty in these inputs propagates to the estimated absorption response. Establishing a device-level detection limit additionally requires validation of the relationship between gas absorption and the measured transmission ratio, together with the complete readout-noise budget. Experimental concentration calibration and noise measurements are therefore needed to assess the projected gas-sensing performance.

## 6. Conclusions

This work presents a numerically evaluated graphene-integrated dual-channel q-BIC metasurface for tunable mid-infrared SO_2_ sensing. Structural symmetry breaking produces two resonances with different modal origins: P1 has an electric-quadrupole-like response and serves as the absorption-sensitive channel, whereas the magnetic-dipole-like P2 resonance provides an off-band reference. In simulation, graphene tuning places P1 at 8.6016 μm for $E_{F}=0.516\;\mathrm{eV}$, enabling spectral alignment after integration of the dielectric and gate layers.

The two channels provide complementary corrections. The P2 shift $\Delta \lambda_{2}$ estimates background refractive-index drift, while the index-dependent baseline $R_{0}\left(n\right)$ corrects the P1/P2 intensity ratio. Under the ideal common-mode model, this ratio gives a 17.03 dB SNR gain at 1% source fluctuation. With η=1 and $n_{\mathrm{eff}}=1$, the field-overlap model gives a conditional LOD of 6.5 ppm under the illustrative rescaled noise budget specified in Section S3, with negligible additional calibration errors. A value of 4.61 ppm would require the total corrected equivalent absorption noise to reach $10^{-7}$. These values are assumption-dependent projections rather than experimentally measured detection limits.

Experimental work is required to determine the accessible graphene tuning range, gas–field interaction length, temperature dependence, complete noise budget, $R_{0}\left(n\right)$ calibration uncertainty, and long-term stability of the proposed sensing architecture.

## Figures and Tables

**Table 1:** Resonance parameters of the integrated graphene-gated metasurface for different gold-grid linewidths ($E_{F}=0\;\mathrm{eV}$).

$w_{\mathrm{Au}}$ (nm)	$\lambda_{P1}$ (μm)	$Q_{P1}$	$\lambda_{P2}$ (μm)	$Q_{P2}$	$T_{\mathrm{base}}$ (%)
100	8.649	456	10.744	456	86.5
200	8.651	397	10.738	411	84.8
500	8.662	263	10.695	213	83.7

**Table 2:** Resonance wavelengths of P1 and P2 under different surrounding refractive indices at the final sensing state ($E_{F}=0.516\mathrm{eV}$).

*n*	$\lambda_{P1}$ (μm)	$\lambda_{P2}$ (μm)
1.00	8.6016	10.7228
1.01	8.6236	10.7280
1.02	8.6460	10.7340
1.03	8.6696	10.7392
1.04	8.6924	10.7448
1.05	8.7152	10.7512
Sensitivity	2280.0 nm RIU^−1^	564.6 nm RIU^−1^
Linearity $R^{2}$	0.9999	0.9991

**Table 3:** Calculated SNR of single-channel and ideal ratiometric readout under different source-fluctuation levels.

$\sigma_{\epsilon }$ (%)	Single (dB)	Ratio (dB)	Gain (dB)
0.1	56.99	56.99	0.00
0.5	45.85	56.99	11.14
1.0	39.96	56.99	17.03
2.0	33.97	56.99	23.02

**Table 4:** Field-overlap-based SO_2_ LOD calculations. All values use η=1 and $n_{\mathrm{eff}}=1$.

Case	Equivalent Absorption-Noise Condition	Calculated LOD
Uncalibrated baseline	$\sigma_{A,\mathrm{uncal}}=1.0\times 10^{-6}$	46.1 ppm
Illustrative rescaled ratio budget	$\sigma_{A,\mathrm{corr}}=1.41\times 10^{-7}$	6.5 ppm ^a^
Total corrected low-noise case	$\sigma_{A,\mathrm{corr}}=1.0\times 10^{-7}$	4.61 ppm

^a^ The source-to-detector noise ratio is s/d=10 and ρ=1. The source fluctuations cancel, leaving the two independent detector contributions. Additional fitting and $R_{0}\left(n\right)$ calibration errors are neglected; the stated absolute noise levels are assumptions.

**Table 5:** Spectral coverage, analytes, sensing mechanisms, and evidence types of related gas and refractive-index sensing approaches.

Study	Band or Analyte	Sensing Mechanism	Evidence and Scope
Present design	8 μm to 12 μm; targeting SO_2_	Gas absorption estimated by field overlap; dual-resonance transmission and reference-assisted ratio	Numerical optical response and conditional LOD model
Meng et al. [47]	7 μm to 14 μm; multiple gases, including NH_3_ and MEK	Metasurface filter array and microbolometer-camera microspectrometer	Experimental system; overlapping band, but different analytes and optical arrangement
Pan et al. [15]	Gas-specific excitation; C_2_H_2_	Acoustic resonator and tuning fork; fiber-optic photoacoustic readout	Experimental transduction architecture; complementary detection mechanism
Wu et al. [16]	Near infrared; C_2_H_2_ and CH4	Acoustic and mechanical resonance enhancement; fiber-optic photoacoustic readout	Experimental multi-gas system; different band and analytes
Yang et al. [17]	C_2_H_2_ demonstration	Multipass optical enhancement and mechanically resonant cantilever; fiber-optic photoacoustic readout	Experimental gas detection; different analyte and detection mechanism

MEK: methyl ethyl ketone. The reported systems differ in analyte, optical path, transduction mechanism, and validation conditions.

## Data Availability

Data will be made available upon reasonable request.

## Supplementary Materials

The following supporting information can be downloaded at: https://www.mdpi.com/article/10.3390/s26216889/s1. Section S1, dual-channel background-index correction; Section S2, ratiometric noise model and imperfect correlation; Section S3, absorption-type LOD inputs, noise budgets and sensitivity analysis; Tables S1–S4; Figure S1; and the archived SO_2_ absorption cross-section file SO_2__sigma_from_HITRAN.csv.
