# Supplementary material for: Graphene-Integrated Dual-Channel q-BIC Metasurface for Actively Tunable and Self-Calibrated Mid-Infrared SO2 Sensing
Source: Sensors (Basel). 2026 Oct 30;26(21):6889. doi: 10.3390/s26216889 (PMC13766505; doi:10.3390/s26216889)
Supplement: Supplementary file 1 [file sensors-26-06889-s001.zip › sensors-4549769-supplementary.pdf]

Supplementary Information: Graphene-integrated dual-channel q-BIC metasurface for actively tunable and self-calibrated mid-infrared SO_2 sensing

Zhengshan Xu[a,b], Dashuai Xu[a,b] , Yang He[a,b] ,Tonggang Zhao[a,b,*]

a Beijing Key Laboratory of Space-Ground Interconnection and Convergence, Beijing University of Posts and Telecommunications, Beijing 100876, China

b School of Electronic Engineering, Beijing University of Posts and Telecommunications, Beijing 100876, China

* Corresponding author: zhaotg@bupt.edu.cn

This supplementary material provides the mathematical basis used in the main manuscript. Section $S1$ derives the dual-channel correction of background refractive-index drift. Section $S2$ derives the ratiometric suppression of common-mode source-intensity noise. Section $S3$ connects the corrected ratio output to the absorption-type limit-of-detection (LOD) model and states the assumptions under which the reported LOD projections are obtained.

The wavelength-based correction estimates and removes background refractive-index drift. It is not used as a direct SO_2 concentration estimator. The concentration and LOD estimates are obtained from the corrected absorption observable A_{corr} and the field-overlap response slope S_A.

S1 Dual-channel correction of background refractive-index drift

At the final sensing state, the graphene Fermi level is fixed at $E_F = 0.516\ eV$. The resonance wavelengths at $n = 1.00$ are $\lambda_{P1} = 8.6016\ \mu m$ and $\lambda_{P2} = 10.7228\ \mu m$. $P1$ overlaps with the SO_2 absorption band and serves as the sensing channel. $P2$ lies outside the main SO_2 absorption band and is used as the off-band reference channel for environmental drift correction.

Let n0=1.00 denote the no-gas baseline refractive index at the fixed final Fermi level EF=0.516 eV, and let $\lambda_{i,0}$ be the corresponding reference wavelength of channel i. The measured wavelength shift is defined as $\Delta\lambda_i = \lambda_i - \lambda_{i,0}$, and $\Delta n = n - n_0$. For small perturbations, the resonance wavelength of channel i can be linearized as

$$\lambda_i(n, \mathbf{q}) \approx \lambda_{i,0} + \left.\frac{\partial \lambda_i}{\partial n}\right|_0 \Delta n + \sum_k \left.\frac{\partial \lambda_i}{\partial q_k}\right|_0 \Delta q_k + \varepsilon_{\lambda,i}$$

Here, q denotes variables other than the background refractive index that may deviate from the baseline state, and $\varepsilon_{\lambda,i}$ is the peak-location or fitting error. The static fabrication offset is included in $\lambda_{i,0}$ after no-gas calibration and is not treated as a time-varying wavelength shift.

$$\delta\lambda_i \equiv \sum_k \frac{\partial\lambda_i}{\partial q_k}\bigg|_0 \Delta q_k + \varepsilon_{\lambda,i} + O(\Delta n^2)$$

Stacking the linearized wavelength-shift equations for P1 and P2 gives

$$\begin{bmatrix}\Delta\lambda_1\\ \Delta\lambda_2\end{bmatrix} = \begin{bmatrix}S_{1,n}\\ S_{2,n}\end{bmatrix}\Delta n + \begin{bmatrix}\delta\lambda_1\\ \delta\lambda_2\end{bmatrix}$$

Equivalently, the two rows of the preceding matrix equation satisfy $\Delta\lambda_i = S_{i,n}\Delta n + \delta\lambda_i,\ i = 1,2$.

Thus, $\delta\lambda_i$ is the residual wavelength shift not described by the linear background-index term. It may include small gate-bias drift, temperature-dependent effects not represented by Δn, residual gas-induced dispersion, model mismatch, and peak-fitting uncertainty. In the present fixed-bias calculation, these terms are treated as residual errors rather than independent sensing observables.

Because P2 is located in a spectral window with weak SO_2 absorption, its non-index residual shift is assumed to be small compared with its background-index-induced shift. The exact reference estimate is

$$\widehat{\Delta n} = \frac{\Delta\lambda_2 - \delta\lambda_2}{S_{2,n}} \approx \frac{\Delta\lambda_2}{S_{2,n}}$$

The approximation on the right is used only when $\delta\lambda_2$ is negligible. Weak SO_2 absorption at P2 supports this approximation but does not make $\delta\lambda_2$ identically zero.

Using the approximation $\widehat{\Delta n} \approx \Delta\lambda_2/S_{2,n}$, the practical P1 wavelength correction is defined as

$$\Delta\lambda_{1,\mathrm{corr}} = \Delta\lambda_1 - \left(\frac{S_{1,n}}{S_{2,n}}\right)\Delta\lambda_2$$

Thus, the correction removes the estimated common linear background-index contribution from P1 but retains residual non-index shifts and peak-fitting uncertainty. It is used for spectral tracking and operating-point correction rather than as a direct SO_2 concentration estimator.

At the final sensing state of $E_F = 0.516\ eV$, $P1$ shifts from $8.6016\ \mu m$ to $8.7152\ \mu m$ as n increases from 1.00 to 1.05. A linear fit yields $S_{1,n} = 2280.0\ nm/RIU$ with $R^2 = 0.9999$. $P2$ shifts from 10.7228 μm to 10.7512 μm, giving $S_{2,n} = 564.6\ nm/RIU$ with $R^2 = 0.9991$. The sensitivity ratio $S_{1,n}/S_{2,n}$ is approximately 4.04.

The refractive-index sweep also shows that the resonance depths vary with n.Therefore, wavelength correction alone is insufficient for an intensity-based gas measurement. The no-gas $P1/P2$ ratio is treated as an index-dependent baseline,

$$R_0(n) = \frac{T_{1,0}(n)}{T_{2,0}(n)}$$

where T1,0(n) and T2,0(n) are the no-gas transmission values of P1 and P2,respectively. Using the P2 wavelength estimate, the baseline is evaluated at $\hat{n} = n_0 + \widehat{\Delta n}$. The measured ratio is $R = I_1/I_2$, and the corrected equivalent absorption observable is defined as

$$A_{\text{corr}} = -\ln\left[\frac{R}{R_0(\hat{n})}\right]$$

For weak SO_2 absorption,

$$A_{\text{corr}} \approx S_A \Delta C$$

Here, $S_A = dA_{corr}/dC$ is the field-overlap absorption-response slope at the final sensing state, and $\Delta C = C - C_0$ is the SO_2 concentration change relative to the no-gas baseline. For $C_0 = 0$, ΔC is equal to the SO_2 concentration, expressed in ppm.

S2 Theoretical Basis of Ratiometric SNR Enhancement

In the present simplified model, temporal source-intensity fluctuation is treated as the dominant common-mode noise component. A multivariate Taylor expansion is used to show how the ratiometric readout suppresses this component to first order.

Noise Model Definition

Let the time-varying light source power be $P(t) = P_0[1 + \varepsilon(t)]$, where P_0 is the average power and $\varepsilon(t)$ is a zero-mean random variable representing relative fluctuation ($\sigma_\varepsilon(t) \ll 1$). The detected signals for both channels are:

$$I_1(t) = P_0[1 + \varepsilon(t)]T_1 + N_1(t)$$
$$I_2(t) = P_0[1 + \varepsilon(t)]T_2 + N_2(t)$$

where T_k is the optical transmittance and $N_k(t)$ is the additive detector noise (uncorrelated white noise with variance σ_{det}^2).

Multivariate Taylor Expansion

Define the ratio output $Y = f(I_1, I_2) = I_1/I_2$. Expanding $f(I_1, I_2)$ to the second order around the mean point (μ_1, μ_2):

$$Y \approx f(\mu_1, \mu_2) + \sum_{i=1}^{2} \frac{\partial f}{\partial I_i}(I_i - \mu_i) + \frac{1}{2}\sum_{i=1}^{2}\sum_{j=1}^{2} \frac{\partial^2 f}{\partial I_i \partial I_j}(I_i - \mu_i)(I_j - \mu_j)$$

The partial derivatives at (μ_1, μ_2) are:

$$\frac{\partial f}{\partial I_1} = \frac{1}{\mu_2}, \quad \frac{\partial f}{\partial I_2} = -\frac{\mu_1}{\mu_2^2}, \quad \frac{\partial^2 f}{\partial I_2^2} = \frac{2\mu_1}{\mu_2^3}, \quad \frac{\partial^2 f}{\partial I_1 \partial I_2} = -\frac{1}{\mu_2^2}$$

Substituting into the expansion (where $\Delta I_k = I_k - \mu_k$):

$$Y \approx \frac{\mu_1}{\mu_2} + \frac{\Delta I_1}{\mu_2} - \frac{\mu_1 \Delta I_2}{\mu_2^2} + \frac{\mu_1(\Delta I_2)^2}{\mu_2^3} - \frac{\Delta I_1 \Delta I_2}{\mu_2^2}$$

Variance Analysis and Covariance Cancellation

For the leading-order variance calculation, only the first-order terms of the Taylor expansion are retained; the second-order terms are treated as higher-order residuals.

The relative variance of Y is approximated as:

$$\frac{\sigma_Y^2}{\bar{Y}^2} \approx \frac{\sigma_{I1}^2}{\mu_1^2} + \frac{\sigma_{I2}^2}{\mu_2^2} - 2\frac{\mathrm{Cov}(I_1, I_2)}{\mu_1 \mu_2}$$

Since both channels share the light source fluctuation $\varepsilon(t)$, they are highly correlated: $\mathrm{Cov}(I_1, I_2) \approx (P_0 T_1)(P_0 T_2)\sigma_\varepsilon^2$. Substituting $\sigma_{I_k}^2 \approx (P_0 T_k)^2 \sigma_\varepsilon^2 + \sigma_{det,k}^2$:

$$\frac{\sigma_Y^2}{\bar{Y}^2} \approx \left[\frac{(P_0 T_1)^2 \sigma_\varepsilon^2 + \sigma_{det1}^2}{\mu_1^2} + \frac{(P_0 T_2)^2 \sigma_\varepsilon^2 + \sigma_{det2}^2}{\mu_2^2}\right] - 2\sigma_\varepsilon^2 = \frac{\sigma_{det1}^2}{\mu_1^2} + \frac{\sigma_{det2}^2}{\mu_2^2}$$

Identical fractional source fluctuations cancel to first order in the ideal common-mode limit. Residual noise depends on detector noise, channel mismatch, imperfect source-noise correlation, higher-order terms, and calibration errors; its dominant contribution must be determined from the actual readout.

Quantification of SNR Enhancement

For the quantitative comparison, the detector-noise standard deviation normalized by the mean signal of each channel is assumed to be $\sigma_d = \frac{\sigma_{\det,k}}{\mu_k} = 1.0 \times 10^{-3}, \quad k = 1,2.$ The two channels are assumed to have equal normalized detector-noise levels, and their detector-noise components are assumed to be mutually uncorrelated. The SNR in decibels is defined as

$$\mathrm{SNR} = -20\log_{10}(\sigma_{\mathrm{rel}})$$

Here σ_rel is the fractional standard deviation of the baseline readout. This inverse-noise convention does not by itself specify the SNR of a gas-induced signal. For the single-channel readout, independent source and detector noise give

$$\mathrm{SNR}_{\mathrm{single}} = -20\log_{10}\left[\sqrt{\sigma_\varepsilon^2 + \sigma_d^2}\right]$$

In the ideal ratiometric limit, the common-mode source fluctuation is canceled to first order, while the uncorrelated detector-noise contributions from the two channels remain. Therefore,

$$\mathrm{SNR}_{\mathrm{ratio}} = -20\log_{10}\left[\sqrt{2\sigma_d^2}\right]$$

These expressions give an ideal ratiometric SNR of 56.99 dB. At $\sigma_\varepsilon = 0.01$ (1.0%), the single-channel SNR is 39.96 dB and the calculated gain is 17.03 dB. Figure S1 shows the full 0–5% source-fluctuation range.

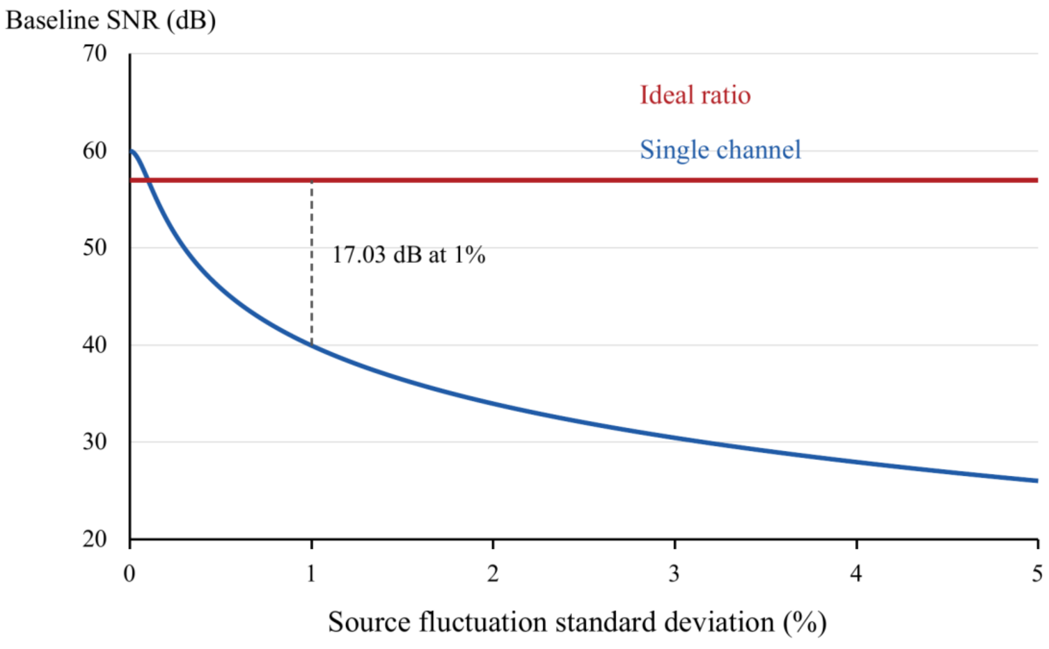

Figure S1. Theoretical SNR comparison between single-channel and ratiometric readout under the ideal common-mode source-noise model.

The blue curve shows the single-channel baseline SNR, and the red curve shows the ideal ratio result. The finite gain of 17.03 dB compares the total single-channel and ratio noise levels, including detector noise. It is not a factor that can be applied separately to an arbitrary common-mode noise component. Fitting and baseline-calibration uncertainties are additional contributions.

Imperfect correlation and unequal fluctuations

Let s_1 and s_2 be the fractional source-noise standard deviations at P1 and P2, ρ their correlation coefficient, and d_1 and d_2 the fractional detector-noise standard deviations. Detector noise is assumed independent between channels and uncorrelated with source noise. For small fluctuations,

$$\sigma_{\ln R}^2 \approx s_1{}^2 + s_2{}^2 - 2\rho s_1 s_2 + d_1{}^2 + d_2{}^2.$$

For equal source fluctuations $s_1 = s_2 = s$ and equal detector noise $d_1 = d_2 = d$, the noise-rejection gain relative to channel 1 is

$$G_{dB} = 10\, log_{10} \frac{s^2 + d^2}{2s^2(1 - \rho) + 2d^2}.$$

Table S1 Effect of source-noise correlation on the calculated gain

Correlation ρ	Ratio noise $\sigma_{\ln R}$	Gain (dB)
1.00	1.414×10^{-3}	17.03
0.99	2.000×10^{-3}	14.02
0.95	3.464×10^{-3}	9.25
0.90	4.690×10^{-3}	6.62

Table S1 uses s = 0.01 and d = 0.001. The gain falls from 17.03 dB at ρ = 1 to 9.25 dB at ρ = 0.95. Even at ρ = 1, unequal source fluctuations leave the residual variance $(s_1 - s_2)^2$. A shared optical path alone does not establish equal fluctuations or perfect correlation. Interpreting the calculated noise rejection as a gas-detection SNR gain also requires an unchanged concentration-response slope and negligible target-gas response in P2. Both channels must use compatible acquisition timing, bandwidth and averaging.

S3 Absorption-type LOD model and noise assumptions

The LOD calculation is based on an absorption-type observable rather than direct subtraction of concentration-dependent FDTD transmission spectra. At ppm-level SO_2 concentrations, the imaginary refractive-index change of the SO_2-air mixture is extremely small. Direct FDTD concentration sweeps therefore produce nearly overlapping transmission spectra and do not provide a numerically robust concentration difference. The absorption response is instead calculated from the molecular absorption coefficient and the simulated gas-field overlap.

The concentration-dependent molecular absorption coefficient is

$$\alpha(\lambda, C) = \sigma(\lambda) N_{air} C_{ppm} \times 10^{-6}$$

Here $\sigma(\lambda)$ is the SO_2 absorption cross section, $N_{air} = 2.46 \times 10^{19}$ molecules cm^{-3} is the assumed air number density, and C_ppm is the concentration in ppm. The archived HITRAN-based cross-section table is used as the numerical input; the database reference is [S1]. The table contains 2001 wavelengths from 8 to 12 μm at 0.002 μm spacing. Linear interpolation at 8.6016 μm gives $\sigma = 4.86867 \times 10^{-20}$ cm^2 $molecule^{-1}$, reproducing the effective absorption slope below.

The resonant field overlap with the gas-accessible region is

$$\Gamma_{gas} = \frac{\int_{V_{SO_2}} |E(\boldsymbol{r})|^2 \, dV}{\int_{V_{all}} |E(\boldsymbol{r})|^2 \, dV}$$

Here, V_{SO_2} includes the side-accessible gas region around the Si pillar and the top gas region above the metasurface, whereas V_{all} is the full monitored volume. The hollow interior is not included because it is not gas-accessible in the present configuration. The side-accessible region is expected to provide the dominant contribution because of the stronger fields near the Si sidewalls and corners.

At the aligned $P1$ resonance, $\Gamma_{gas} = 0.7826$. The side-accessible gas region contributes $\Gamma_{side} = 0.5640$, and the top gas region contributes $\Gamma_{top} = 0.2185$.

The effective absorption coefficient is

$$\alpha_{eff}(C) = \Gamma_{gas} \alpha(C)$$

with the concentration slope

$$\frac{d\alpha_{eff}}{dC} = 9.37 \times 10^{-7} \, cm^{-1} \, ppm^{-1}$$

The effective interaction length is estimated as

$$L_{eff} = \eta \frac{Q \lambda_{P1}}{2\pi n_{eff}}$$

The baseline projection uses $\eta = 1$ and $n_{eff} = 1$. These values are idealized normalization factors, not independently measured modal parameters. With $Q = 506.86$ and $\lambda_{P1} = 8.6016 \, \mu m$,

$$L_{eff} \approx 6.94 \times 10^{-2} \, cm = 693.9 \, \mu m$$

The equivalent absorption-response slope is therefore

$$S_A = L_{eff} \frac{d\alpha_{eff}}{dC} \approx 6.50 \times 10^{-8} \, ppm^{-1}$$

Table S2 Inputs and derived quantities for the field-overlap projection

Quantity	Value	Basis
Operating point	E_F = 0.516 eV; λ_{P1} = 8.6016 µm; Q = 506.86	Final sensing state and spectrum-derived Q reported in Section 3.4 of the main article
Gas-field overlap	Γ_{gas} = 0.7826	Gas-field overlap reported in Section 4.4 and Figure 7(d) of the main article; gas-accessible volume defined in Section S3
SO_2 cross section at P1	4.86867 × 10^{-20} cm^2 $molecule^{-1}$	Linear interpolation of the archived cross-section table
Air number density	2.46 × 10^{19} molecules cm^{-3}	Model normalization
Effective absorption slope	9.37 × 10^{-7} cm^{-1} ppm^{-1}	Γ_{gas} σ(λ_{P1}) N_{air} × 10^{-6}
Interaction normalization	η = 1; n_{eff} = 1	Idealized assumptions, not measured modal parameters
Effective interaction length	693.9 µm	η Q λ_{P1}/(2π n_{eff})
Absorption-response slope	6.50 × 10^{-8} ppm^{-1}	L_{eff} $d\alpha_{eff}/dC$

The numerical cross-section input is archived as SO2_sigma_from_HITRAN.csv in the accompanying Supplementary_Data directory. It is supplied directly so that the interpolation and the LOD arithmetic do not require regeneration of a line spectrum. The assumed number density is a separate concentration normalization. The overlap uses the $|E|^2$ integral over the monitored volume, rather than a permittivity-weighted electromagnetic energy fraction. The interaction length remains a Q-based approximation; η and n_{eff} retain its coupling and modal-normalization dependence.

The detection limit is calculated as

$$LOD = \frac{3\sigma_A}{S_A}$$

For an uncalibrated equivalent absorption noise $\sigma_{A,uncal} = 1.0 \times 10^{-6}$,

$$LOD_{uncal} \approx 46.1\, ppm$$

Noise budgets and conditional LOD values

For an experimentally calibrated baseline, the total variance of $A_{corr} = -\ln[R/R_0(\hat{n})]$ also contains index-estimation and baseline-fit errors. To first order,

$$\sigma_A^2 \approx \sigma_{\ln R}^2 + b^2\, u^2(\hat{n}) + u_{cal}^2 - 2b\, Cov(\ln R, \hat{n}),$$

where $b = \partial \ln R_0/\partial n$ at the operating point. This expression assumes that the remaining baseline-fit error is independent of ln R and $\hat{n}$; otherwise further covariance terms are required. Peak-fitting errors used to estimate $\hat{n}$ enter through $u(\hat{n})$ and must not be counted twice. The measured no-gas baseline must include stable source-spectrum and channel-responsivity factors, in addition to the simulated transmission ratio.

The 17.03 dB example in Section S2 uses s = 0.01 and d = 0.001 and gives $\sigma_{\ln R} = 1.414 \times 10^{-3}$ at $\rho = 1$. Its dimensionless gain alone does not establish an absolute absorption-noise floor of 10^{-7}. To illustrate a 6.5 ppm projection consistently, a separate hypothetical noise budget preserves s/d = 10 while setting the total single-channel noise to 10^{-6}:

$$s = \frac{10 \times 10^{-6}}{\sqrt{101}} = 9.9504 \times 10^{-7}; \quad d = \frac{10^{-6}}{\sqrt{101}} = 9.9504 \times 10^{-8}.$$

For $s_1 = s_2 = s$, $d_1 = d_2 = d$, $\rho = 1$ and negligible additional calibration errors, the source term cancels and the two independent detector contributions remain:

$$\sigma_{A,corr} = \sqrt{2}d = 1.4072 \times 10^{-7}; \quad LOD \approx 6.5\,ppm.$$

The corresponding total-noise ratio is $\sqrt{[(s^2 + d^2)/(2d^2)]} = 7.1063$, or 17.03 dB. This is an illustrative rescaling of the assumed noise budget, not a measured noise reduction or a noise floor obtained from FDTD. The 4.61 ppm case separately assumes a total corrected absorption-noise standard deviation of 10^{-7}.

Table S3 Conditional LOD values under explicitly assumed noise budgets

Case	Equivalent absorption noise	LOD (ppm)
Single-channel baseline	Total $\sigma_A = 1.0 \times 10^{-6}$	46.1
Illustrative rescaled ratio budget	Total $\sigma_A = 1.4072 \times 10^{-7}$; assumptions stated above	6.5
Specified lower total-noise case	Total $\sigma_A = 1.0 \times 10^{-7}$	4.61

All cases use $\eta = 1$ and $n_{eff} = 1$. Each noise standard deviation refers to the equivalent absorption observable after the stated readout and processing; it is not a spectral density. Any experimental comparison must specify bandwidth and averaging. These conditional field-overlap estimates are not experimentally measured gas-detection limits.

Sensitivity and uncertainty propagation

The LOD scales linearly with σ_A and inversely with the magnitude of S_A. For the present interaction-length model,

$$LOD(\eta, n_{eff}) = LOD(1,1)\,\frac{n_{eff}}{\eta}.$$

Table S4 Deterministic sensitivity of the LOD to model inputs

Change with other inputs fixed	LOD multiplier
σ_A increases by 20%	1.20
$\lvert S_A \rvert$ decreases by 20%	1.25
$\lvert S_A \rvert$ increases by 20%	0.833
n_{eff} increases by 20%	1.20
η decreases by 20%	1.25

For small uncertainties, let D denote LOD and $B = |S_A|$. First-order propagation including covariance gives [S2]

$$\frac{u^2(D)}{D^2} \approx \frac{u^2(\sigma_A)}{\sigma_A^2} + \frac{u^2(B)}{B^2} - \frac{2\,Cov(\sigma_A, B)}{\sigma_A\, B}.$$

Here $u(\sigma_A)$ is the uncertainty in the estimated noise standard deviation; it is distinct from σ_A itself. Independent inputs remove the covariance term. Field overlap, gas-accessible volume, cross-section input and interaction-length normalization contribute through S_A. Table S4 is a sensitivity calculation, not an experimentally determined confidence interval. Quantitative uncertainty bounds require uncertainties and covariances for the actual inputs.

Supplementary references

[S1] Gordon, I.E.; Rothman, L.S.; Hargreaves, R.J.; Hashemi, R.; Karlovets, E.V.; Skinner, F.M.; Conway, E.K.; Hill, C.; Kochanov, R.V.; Tan, Y.; et al. The HITRAN2020 molecular spectroscopic database. J. Quant. Spectrosc. Radiat. Transf. 2022, 277, 107949. https://doi.org/10.1016/j.jqsrt.2021.107949.

[S2] Joint Committee for Guides in Metrology. Evaluation of Measurement Data—Guide to the Expression of Uncertainty in Measurement; JCGM 100:2008; BIPM: Sèvres, France, 2008. https://doi.org/10.59161/JCGM100-2008E.
